# Evidence for functional convergence in genes upregulated by herbivores ingesting plant secondary compounds

**DOI:** 10.1186/1472-6785-14-23

**Published:** 2014-08-15

**Authors:** Jael R Malenke, Michele M Skopec, M Denise Dearing

**Affiliations:** 1 Department of Biology, 257 South 1400 East, University of Utah, Salt Lake City, UT 84112, USA; 2 Department of Zoology, 1415 Edvalson Street, Weber State University, Ogden, UT 84408, USA

**Keywords:** Herbivory, Diet switching, Mammalian herbivores, *Neotoma*, Biotransformation, Microarray

## Abstract

**Background:**

Nearly 40 years ago, Freeland and Janzen predicted that liver biotransformation enzymes dictated diet selection by herbivores. Despite decades of research on model species and humans, little is known about the biotransformation mechanisms used by mammalian herbivores to metabolize plant secondary compounds (PSCs). We investigated the independent evolution of PSC biotransformation mechanisms by capitalizing on a dramatic diet change event—the dietary inclusion of creosote bush (*Larrea tridentata*)—that occurred in the recent evolutionary history of two species of woodrats (*Neotoma lepida* and *N. bryanti*).

**Results:**

By comparing gene expression profiles of two populations of woodrats with evolutionary experience to creosote and one population naïve to creosote, we identified genes either induced by a diet containing creosote PSCs or constitutively higher in populations with evolutionary experience of creosote. Although only one detoxification gene (an aldo-keto reductase) was induced by both experienced populations, these populations converged upon functionally equivalent strategies to biotransform the PSCs of creosote bush by constitutively expressing aldehyde and alcohol dehydrogenases, Cytochromes P450s, methyltransferases, glutathione S-transferases and sulfotransferases. The response of the naïve woodrat population to creosote bush was indicative of extreme physiological stress.

**Conclusions:**

The hepatic detoxification system of mammals is notoriously complex, with hundreds of known biotransformation enzymes. The comparison herein of woodrat taxa that differ in evolutionary and ecological experience with toxins in creosote bush reveals convergence in the overall strategies used by independent species after a historical shift in diet. In addition, remarkably few genes seemed to be important in this dietary shift. The research lays the requisite groundwork for future studies of specific biotransformation pathways used by woodrats to metabolize the toxins in creosote and the evolution of diet switching in woodrats. On a larger level, this work advances our understanding of the mechanisms used by mammalian herbivores to process toxic diets and illustrates the importance of the selective relationship of PSCs in shaping herbivore diversity.

## Background

For herbivorous animals, food ingestion can have deleterious consequences [1,2]. At every meal, herbivores confront potential toxins in the form of plant secondary compounds (PSCs). Some herbivores, e.g., the black swallowtail (*Papilio polyxenes*) and Stephen’s woodrat (*Neotoma stephensi*), have evolved the ability to specialize on plant species with high concentrations of PSCs, whereas other herbivores must consume undefended plants or small quantities of many species of plants with differing PSCs to keep toxin intake below threshold doses [3-6].

In general, diet selection and diet breadth of herbivores is thought to be governed by the capacity of biotransformation (detoxification) enzymes in the liver [7,8]. Major evolutionary dietary shifts seem to be enabled by an underlying change in biotransformation enzymes [9,10]. Surprisingly few alterations among myriad biotransformation enzymes are required for a change in the capacity to metabolize a new toxin. Insect resistance to pesticides and mammalian resistance to rodenticides can occur through the increased activity of a single biotransformation enzyme [9,11]. Currently, far more is known about the biotransformation mechanisms employed by insect herbivores in a diet shift than their mammalian counterparts [1,9,12].

The vast diversity of biotransformation enzymes presents a challenge in determining which enzymes enable the processing of a particular diet or toxin. For example, more than 300 hepatic biotransformation enzymes have been described for the laboratory rat, *Rattus norvegicus* (http://www.reactome.org, [13]). Enzyme activity assays exist for far fewer than the known number of biotransformation enzymes and are not always specific to a gene product [14]. To overcome these challenges, we took a genomic approach and used microarray technology customized for our study species, to identify, on a more global scale, biotransformation gene expression patterns involved in the processing of PSCs. Gene expression is an important phenotypic character, subject to selection from the environment [14-16]. Many recent studies have concluded that differences in gene expression among groups are the result of disparate selective forces [17-19].

We investigated the independent evolution of biotransformation mechanisms related to diet switching by capitalizing on a dramatic diet change event that occurred in the recent evolutionary history two species of woodrats (*Neotoma lepida*, *N. bryanti*). Both species underwent radical dietary changes due to floral shifts during the climate change event of the late Pleistocene and early Holocene (18,700-10,000 years ago). This event resulted in both of these woodrats independently switching from diets of juniper and/or cactus, to one containing creosote bush as it naturally invaded from Mexico and expanded to become the dominant shrub in the major deserts of the U.S. southwest [20,21]. Fossil records of middens of both species indicate that *N. bryanti* came into contact with creosote 7,000 years before *N. lepida*[22,23].

The change in diet to creosote from previous plant species represents a marked change in PSC composition. Juniper contains high concentrations of numerous terpenes as well as less abundant tannins [24,25]. The primary PSC in cactus (oxalate) is only degraded by gut microbes, not liver enzymes [26]. In contrast, creosote leaves produce a complex resin containing >300 compounds, primarily aromatic ones, which account for 10-25% of the leaf dry weight [27]. The principal component of resin is nordihydroguaiaretic acid (NDGA), a phenolic lignan with detrimental effects when administered to laboratory rats in doses regularly consumed by woodrats [28-30]. The distinct differences in the PSCs of juniper and cactus versus creosote suggest they are metabolized by different biotransformation pathways [14,31]. Comparative data on enzyme activities and gene expression of populations of *N. lepida* support this contention. *Neotoma lepida* that feed on juniper have different enzyme activity and gene expression profiles compared to those that feed on creosote even when they are fed the same diet [32-34]. Indeed, populations of *N. lepida* in the Mojave desert have adapted to a diet of creosote as evidenced by their ability to ingest greater quantities of creosote compared to those from the Great Basin desert that feed on juniper and have no prior exposure to creosote [35]. Less is known about the ancestral diet of *N. bryanti* and no data exist with respect to its ability to metabolize PSCs. However, a recent comparative study on gene expression in *N. lepida* and *N. bryanti* on a non-toxic diet revealed similarities in expression profiles in populations of both species that feed on creosote, relative to *N. bryanti* populations naïve to creosote [36]. These results are consistent with potential convergence in biotransformation strategies of *N. lepida* and *N. bryanti* with respect to metabolism of a creosote diet.

To advance our knowledge of the mechanisms involved in diet switching in mammals as well as biotransformation of PSCs in general, we addressed three questions. We asked which genes are induced (upregulated) by woodrats in response to the addition of creosote PSCs to the diet. Gene induction is a conventional pharmacological approach used to identify potentially important enzymes in biotransformation of foreign compounds. Second, we asked whether experience mattered with respect to the biotransformation of creosote. We contrasted the responses of woodrats that historically and currently consume creosote to a population that is ecologically and evolutionarily naïve to creosote compounds. Lastly, we asked whether there was convergence in the pathways used for biotransformation of creosote resin between *N. bryanti* and *N. lepida* that regularly fed on creosote bush by comparing the induced transcripts and also by contrasting their gene expression profiles.

## Results

### Feeding trial

All three populations maintained weight on both the control and creosote diet (Additional file 1: Table S1). However, there was a significant difference across populations with the experienced *N. bryanti* being heavier than the other two populations.

### Microarray quality control

Four of the 24 arrays failed to pass all 9 of Agilent’s quality metrics. Three of these passed 8 of 9 metrics, and 1 passed 7 of 9. All were kept in the analysis. On average, less than 0.3% of the features across all arrays were flagged as non-uniform. The clustergram grouping individual woodrats by overall expression profile (all 6286 genes) grouped all the *N. lepida* in one monophyletic clade and each *N. bryanti* population in its own monophyletic clade, but did not group individuals by diet within species designations (Additional file 2: Figure S1).

### Transcripts induced by creosote

Both species and all populations altered gene expression in response to the creosote diet compared to the control. The experienced *N. bryanti* had fewer induced (n = 7) and repressed transcripts (n = 7) than either the experienced *N. lepida* (induced n = 26, repressed n = 20) or the naïve *N. bryanti* (induced n = 18, repressed n = 9); however, the difference among the groups was not significant (Χ^2^ = 1.23 p = 0.54). There were no induced transcripts shared by all groups in this analysis. The experienced and naïve populations of *N. bryanti* shared two induced transcripts, one of which was for a biotransformation gene (aldo-keto reductase 7A3, Table 1). None of the treatment groups on creosote induced more than five different biotransformation genes (Table 1). The largest fold induction of a biotransformation transcript was observed in the naïve *N. bryanti*, which induced sulfotransferase 3A1 by 22×. All other biotransformation transcripts across all groups exhibited inductions of 7-fold or less.

**Table 1:** **Transcripts induced by creosote diet in three treatments: A. naïve *N. bryanti*, B. experienced *N. bryanti and *** **C. experienced *N. lepida***

	**Gene ID**	**Gene description**	**Ratio**	**p-value**
**A.**	Induced in *N. bryanti* – naïve			
	NM_020565	**Sulfotransferase family 3A, member 1**	22.34	0.024497169
	NM_021391	Protein phosphatase 1, regulatory (inhibitor) subunit 1A	12.42	0.005341544
	NM_173295	**UDP glucuronosyltransferase 2 family, polypeptide B17**	3.3	0.01004607
	NM_027153	Pirin, mRNA	3.18	0.049005275
	NM_173295	**UDP glucuronosyltransferase 2 family, polypeptide B17**	2.95	0.005109673
	NM_007631	Cyclin D1	2.75	0.047341015
	NM_010145	**Epoxide hydrolase 1, microsomal**	2.51	0.004864803
	NM_008761	*FXYD domain-containing ion transport regulator 5 transcript variant 2	2.51	0.005930014
	NM_019144	Acid phosphatase 5, tartrate resistant	2.38	0.030204487
	NM_016740	S100 calcium binding protein A11	2.37	0.002381142
	NM_019693	HLA-B-associated transcript 1A	2.31	0.002135102
	NM_013215	**Aldo-**keto reductase family 7, member A3**	2.2	0.048796617
	NM_009673	Annexin A5	2.11	0.022303224
	NM_010664	Keratin 18	2.08	0.034267738
	NM_028070	AlkB, alkylation repair homolog 4 (E. coli)	2.07	0.026611663
	NM_013899	Translocase of inner mitochondrial membrane 10 homolog (yeast)	2.04	0.001531745
	NM_013058	Inhibitor of DNA binding 3	2.03	0.014931095
	NM_001111030	Activin A receptor, type IC	2.02	0.015088922
**B.**	Induced in *N. bryanti* – experienced			
	NM_053346	Neuritin 1	3.99	0.016579939
	NM_001164627	Rho GTPase activating protein 8 transcript variant 1, MutualBestHitTo	3.1	0.0205116
	NM_013215	**Aldo-**keto reductase family 7, member A3**	2.93	0.005358317
	NM_001109171	Leucine rich repeat containing 20	2.65	0.001865456
	NM_008761	*FXYD domain-containing ion transport regulator 5 transcript variant 2	2.23	0.023632655
	NM_027582	RIKEN cDNA 4921521 F21 gene	2.11	0.026277911
	NM_001106470	Similar to KIAA1627 protein	2.05	0.027174423
**C.**	Induced in *N. lepida* – experienced			
	NM_027406	**Aldehyde dehydrogenase 1 family, member L1**	6.9	0.032217115
	NM_029662	Major facilitator superfamily domain containing 2	5.01	0.009297156
	NM_027406	**Aldehyde dehydrogenase 1 family, member L1**	4.5	0.023937762
	NM_001014058	Ubiquitin specific peptidase 18	3.56	0.02525724
	NM_001184980	**Sulfotransferase family 2A DHEA**-**preferring member 5, SimilarTo**	3.43	0.016254811
	NM_031004	Smooth muscle alpha-actin	3.37	0.025794253
	NM_001126273	AlkB, alkylation repair homolog 2 (E. coli)	3.26	0.047075352
	NM_177200	SV2 related protein homolog (rat)-like	3.25	0.007639427
	NM_010145	**Epoxide hydrolase 1, microsomal**	2.99	0.013289403
	NM_178686	Centrosomal protein 120	2.92	0.012963163
	NM_010358	**Glutathione S**-**transferase, mu 1**	2.8	0.022522125
	NM_001168541	Tsukushin transcript variant 1, MutualBestHitTo	2.75	0.007592024
	NM_022331	Ubiquitin-like domain member 1	2.64	0.004966041
	NM_001184980	**Sulfotransferase family 2A DHEA**-**preferring member 5, SimilarTo**	2.45	0.036858859
	NM_031768	Integrin, alpha E, epithelial-associated	2.4	0.035330416
	NM_198780	Phosphoenolpyruvate carboxykinase 1 (soluble)	2.4	0.047233237
	NM_011393	Solute carrier family 1, member 2, transcript variant 3	2.37	0.000661494
	NM_145123	Cartilage acidic protein 1	2.27	0.028029577
	NM_133626	Ribosome binding protein 1	2.27	0.042370631
	NM_029494	RAB30, member RAS oncogene family	2.19	0.030712931
	NM_153392	Tetratricopeptide repeat domain 39A, transcript variant 2	2.15	0.044515732
	NM_138953	Elongation factor RNA polymerase II 2	2.13	0.017500695
	NM_022602	Pim-3 oncogene	2.06	0.018759684
	NM_053433	**Flavin containing monooxygenase 3**	2.03	0.01753571
	NM_028116	Pygopus 1	2.02	0.002651983
	NM_021390	Zinc finger protein Sall1	2.0	0.006073039

Bolded entries have known detoxification function. Asterisks indicate transcripts that are induced in both categories A and B. There are no shared induced transcripts between B and C.

### Differential expression between naïve and experienced woodrats

There were significant differences in expression related to experience with creosote between *N. bryanti* populations. Although the total number of transcripts with greater expression in both the experienced and naïve was similar (199 in experienced vs. 183 naïve, [Table 2A with list of genes in Additional file 3: Table S2]), the experienced population expressed absolutely twice as many transcripts for biotransformation enzymes (Table 2A, Χ^2^ = 14.72, df = 1, p < 0.001). Proportionally, 37% of all transcripts expressed to a greater degree by experienced *N. bryanti* were biotransformation related compared to only 18% of those in the naïve *N. bryanti*. Many of the differentially regulated biotransformation transcripts coded for the same Genbank accession. The effective gene numbers, calculated from Shannon’s H, reflected the same pattern as the total biotransformation transcript counts. For naïve *N. bryanti* the effective gene number was 19.7 compared to an effective gene number for experienced *N. bryanti* of 34.8.

**Table 2:** **Number of transcripts with significantly different expression in a comparison of (A) naïve and experienced *N. bryanti *** **on the creosote diet and (B) experienced *N. bryanti *** **and *N. lepida *** **on the creosote diet**

**A.**		**Transcripts**		
Treatment population		All	Detox	Chi-square
	Higher expression, *N. bryanti*, naïve	183	35	Χ^2^ = 11.92, df = 1, p < 0.001
	Higher expression, *N. bryanti*, experienced	199	76	
**B.**				
Treatment species		All	Detox	Chi-square
	Higher expression, *N. bryanti*, experienced	134	69	Χ^2^ = 14.72, df = 1, p = 0.0001
	Higher expression, *N. lepida*, experienced	109	23	

Chi square analyses compare the number of detoxification genes with higher expression to the overall number of genes with higher expression across the two experimental groups.

The degree to which upregulated genes were expressed was greater, overall, in the experienced *N. bryanti* (Table 3, Additional file 3: Table S2). The biotransformation transcript with the greatest expression in naïve *N. bryanti* and the only one with more than 10-fold higher expression in naïve compared to experienced animals, was a cytochrome P450 (2C65). In contrast, there were three biotransformation transcripts in the experienced *N. bryanti* that exceeded 10-fold higher expression compared to naïve *N. bryanti*. These transcripts are related to the functionalization of aldehydes (aldo-keto reductase 1C12) and glucuronic acid and glutathione conjugation pathways (UDP glucuronosyltransferase 2B34, glutathione S-transferase, mu 7).

**Table 3:** **Transcripts associated with detoxification function with significantly greater expression in (A) naive *N. bryanti *** **relative to experienced *N. bryanti *** **and (B.) experienced *N. bryanti *** **relative to naïve *N. bryanti***

	**Gene ID**	**Gene description**	**Ratio**	**p-value**
**A.**	Greater expression in naïve *N. bryanti*			
		**Phase I - catalyze oxidation**, **reduction and hydrolysis reactions**		
	**Alcohol dehydrogenases - oxidize alcohols**			
	NM_009626	C57BL/6 J alcohol dehydrogenase class 4	2.11	0.019591
	**Carboxylesterases - hydrolyze carboxylic acid esters**			
	NM_145603	Carboxylesterase 2	2.52	0.001284
	NM_145603	Carboxylesterase 2	2.52	0.001213
	NM_021456	Carboxylesterase 1	2.45	0.036796
	NM_021456	Carboxylesterase 1	2.36	0.040035
	NM_172759	Carboxylesterase 5,	2.12	0.001463
	**Cytochromes P450 - oxidize wide range of organic substrates**			
	NM_028191	Cytochrome P450, family 2, subfamily c, polypeptide 65	15.62	0.006602
	NM_007825	Cytochrome P450, family 7, subfamily b, polypeptide 1	4.48	0.000458
	XM_219933	PREDICTED: P450 family 2 subfamily c polypeptide 79, SimilarTo	4.43	0.001591
	NM_028191	Cytochrome P450, family 2, subfamily c, polypeptide 65	3.79	0.009243
	NM_007825	Cytochrome P450, family 7, subfamily b, polypeptide 1,	3.24	0.001260
	NM_019138	Cytochrome P450, family 7, subfamily b, polypeptide 1	2.46	0.002067
	NM_019138	Cytochrome P450, family 7, subfamily b, polypeptide 1	2.2	0.002338
	NM_010009	25-hydroxyvitamin D3 1alpha-hydroxylase	2.12	0.021096
	**Flavin containing monooxygenases - oxidize amines**			
	NM_018881	Flavin containing monooxygenase 2, mRNA	2.78	0.018212
	NM_018881	Flavin containing monooxygenase 2, mRNA	2.66	0.026835
		**Phase II - catalyze transfer of conjugates to metabolites**		
	**Acetyltransferases - transfer acetyl conjugate**			
	NM_001161712	Glycine C-acetyltransferase transcript variant 2 SimilarTo	3.45	0.015595
	**UDP glucuronosyltransferases - transfer glucuronic acid conjugate**			
	NM_172881	UDP glucuronosyltransferase 2 family, polypeptide B35	3.93	0.048799
	NM_153598	UDP glucuronosyltransferase 2 family, polypeptide B34	2.86	0.000686
	NM_153598	UDP glucuronosyltransferase 2 family, polypeptide B34	2.6	0.000899
	NM_001029867	UDP glucuronosyltransferase 2 family, polypeptide B36	2.02	0.000218
	**Glutathione S**-**transferases - transfer glutathione conjugate**			
	NM_012577	Glutathione S-transferase pi	2.67	0.003827
	**Methyltransferases - transfer methyl group conjugate**			
	NM_016785	Thiopurine methyltransferase	5.2	0.000140
	NM_016785	Thiopurine methyltransferase	5.04	0.000190
	NM_016785	Thiopurine methyltransferase	4.72	0.000223
	NM_016785	Thiopurine methyltransferase	4.3	0.000073
	NM_022884	Betaine-homocysteine methyltransferase 2	3.48	0.001257
	NM_022884	Betaine-homocysteine methyltransferase 2	2.15	0.005312
	NM_026440	RNA (guanine-7-) methyltransferase	2.12	0.004311
	NM_022884	Betaine-homocysteine methyltransferase 2	2.11	0.005705
	NM_177846	MKIAA0547 protein	2.04	0.000481
	**Sulfotransferases - tranfer sulfo group conjugate**			
	NM_001184980	Sulfotransferase family 2A DHEA-preferring member 5, SimilarTo	4.75	0.006821
	NM_001101586	Sulfotransferase family 2A DHEA-preferring member 5, SimilarTo	4.73	0.020208
	NM_001101586	Sulfotransferase family 2A DHEA-preferring member 5, SimilarTo	4.69	0.025323
	NM_001101534	CDNA clone IMAGE:9053718	4.11	0.031920
**B.**	Greater expression in experienced *N. bryanti*			
		**Phase I - catalyze oxidation**, **reduction and hydrolysis reactions**		
	**Aldo**-**keto reductases - oxidize and reduce aldehydes and ketones**			
	NM_013777	Aldo-keto reductase family 1, member C12	22.98	0.000139
	NM_030611	Aldo-keto reductase family 1, member C6	7.65	0.000074
	**Aldehyde dehydrogenases - oxidize aldehydes**			
	NM_153543	Aldehyde dehydrogenase 1 family, member L2	3.61	0.000215
	NM_031057	Aldehyde dehydrogenase 6 family, member A1	2.19	0.046613
	**Carboxylesterases - hydrolyze carboxylic acid esters**			
	NM_145603	Carboxylesterase 2	6.91	0.000264
	NM_001190346	Carboxylesterase 2 transcript variant 2, SimilarTo	6.55	0.000143
	NM_145603	Carboxylesterase 2	6.21	0.000575
	NM_001044258	Similar to Carboxylesterase 2	6.04	0.000027
	NM_001044258	Similar to Carboxylesterase 2	5.68	0.000028
	NM_001190346	Carboxylesterase 2, transcript variant 2, SimilarTo	3.63	0.000532
	NM_172759	Carboxylesterase 5	2.9	0.001251
	**Cytochromes P450 - oxidize wide range of organic substrates**			
	NM_023025	Cytochrome P450, family 2, subfamily J, polypeptide 4	6.7	0.002710
	NM_012730	Cytochrome P450, family 2, subfamily d, polypeptide 2	3.38	0.035947
	NM_012730	Cytochrome P450, family 2, subfamily d, polypeptide 2	3.34	0.019677
	NM_012730	Cytochrome P450, family 2, subfamily d, polypeptide 2	3.25	0.047435
	NM_153312	Cytochrome P450, family 3, subfamily a, polypeptide 23/polypeptide 1	3.1	0.006788
	NM_153312	Cytochrome P450, family 3, subfamily a, polypeptide 23/polypeptide 1	2.92	0.000334
	NM_022434	Cytochrome P450, family 4, subfamily f, polypeptide 14	2.34	0.002319
	**Flavin containing monooxygenases - oxidize amines**			
	NM_001161765	Flavin containing monooxygenase 5 transcript variant 1, SimilarTo	2.46	0.005855
	NM_001161765	Flavin containing monooxygenase 5 transcript variant 1, SimilarTo	2.4	0.006221
	**Miscellaneous Phase I**			
	NM_013626	Peptidylglycine alpha-amidating monooxygenase	2.23	0.005322
	NM_001004086	Paraoxonase 3	2.18	0.000331
		**Phase II - catalyze transfer of conjugates to metabolites**		
	**Acetyltransferases - transfer acetyl conjugate**			
	NM_053853	N-acetyltransferase 1	7.05	0.000017
	NM_001108278	Spermidine/spermine N1-acetyltransferase family member 2	6.26	0.000021
	NM_053853	N-acetyltransferase 1	5.39	0.000007
	NM_053853	N-acetyltransferase 1	4.99	0.000010
	NM_001006995	Acetyl-Coenzyme A acetyltransferase 2	3.73	0.004937
	NM_001009657	Histone acetyltransferase 1	2.24	0.000395
	NM_001009657	Histone acetyltransferase 1	2.04	0.000004
	**UDP glucuronosyltransferases - transfer glucuronic acid conjugate**			
	NM_153598	UDP glucuronosyltransferase 2 family, polypeptide B34	11.7	0.000347
	NM_153598	UDP glucuronosyltransferase 2 family, polypeptide B34	7.22	0.013582
	NM_201642	UDP glucuronosyltransferase 1 family, polypeptide A6B	5.21	0.000008
	NM_152811	UDP glucuronosyltransferase 2 family, polypeptide B1	3.37	0.043313
	NM_153598	UDP glucuronosyltransferase 2 family, polypeptide B34	2.24	0.004634
	NM_001191676	UDP glucuronosyltransferase 2 family polypeptide B34, SimilarTo	2.18	0.005383
	NM_153598	UDP glucuronosyltransferase 2 family, polypeptide B34	2.06	0.007980
	**Glutathione S-transferases - transfer glutathione conjugate**			
	NM_026672	Glutathione S-transferase, mu 7	10.47	0.038865
	NM_026672	Glutathione S-transferase, mu 7	9.14	0.034704
	NM_133994	Glutathione S-transferase, theta 3	8.12	0.003689
	NM_133994	Glutathione S-transferase, theta 3	7.66	0.002784
	NM_008183	Glutathione S-transferase, mu 2	4.77	0.000073
	NM_008183	Glutathione S-transferase, mu 2	2.68	0.015619
	NM_001024361	Similar to Glutathione S-transferase A1	2.54	0.040554
	XM_001473911	PREDICTED: Glutathione S-transferase Mu 2, SimilarTo	2.41	0.000974
	NM_001024304	Glutathione S-transferase mu 4	2.11	0.026064
	NM_008183	Glutathione S-transferase, mu 2	2.09	0.001695
	NM_001009920	Glutathione S-transferase Yc2 subunit	2.04	0.031287
	**Methyltransferases - transfer methyl group conjugate**			
	NM_001008299	RNA (guanine-7-) methyltransferase	6.35	0.000009
	NM_022884	Betaine-homocysteine methyltransferase 2	3.45	0.001721
	NM_001106470	Similar to KIAA1627 protein	3.35	0.002862
	NM_010321	Glycine N-methyltransferase	3.26	0.000333
	NM_173765	Aminoadipate-semialdehyde dehydrogenase	3.02	0.000019
	NM_010321	Glycine N-methyltransferase	2.84	0.000468
	NM_027334	Methyltransferase like 7A	2.73	0.002067

All animals were fed a creosote diet. Within populations, results are organized by major detoxification enzyme class. Duplicates indicate the response of multiple probes for a given gene.

Significant Gene Ontology (GO) terms (within biological process) were different in the naïve versus experienced *N. bryanti*. Before comparison, terms across ontology tiers were filtered to include only those with significant, positive z-scores (>2) and ordered by the number of genes in each ontology term. GO terms are reported only if they include 10 or more genes from the array. Naïve *N. bryanti* had more than twice the number of GO terms that passed these criteria. The top terms related to responses to stimuli; many of the other terms related to stress, damage and cell death (Table 4A). The experienced *N. bryanti* had fewer significant terms; the function of these related largely to metabolic processes (Table 4B).

**Table 4:** **Gene ontology** (**GO**) **terms overrepresented in (A) naïve *N. bryanti *** **and (B) experienced *N. bryanti *** **on creosote diets**

**Ontology**	**List**	**Gene set**	**z-score**
**A. naïve *N. bryanti***			
response to stimulus	61	1241	3.66
response to chemical stimulus	30	533	3.13
system development	28	587	2.06
response to stress	27	525	2.44
immune system process	22	265	4.62
apoptosis	20	350	2.56
cell death	20	367	2.34
death	20	370	2.3
programmed cell death	20	353	2.52
regulation of multicellular organismal process	20	328	2.88
regulation of apoptosis	17	287	2.51
regulation of cell death	17	297	2.35
regulation of programmed cell death	17	289	2.47
cellular response to chemical stimulus	15	222	2.89
immune response	15	140	4.92
regulation of developmental process	15	244	2.5
response to external stimulus	13	189	2.75
regulation of multicellular organismal development	12	206	2.02
defense response	11	147	2.83
positive regulation of developmental process	11	113	3.82
response to wounding	11	137	3.09
hemopoiesis	10	103	3.63
hemopoietic or lymphoid organ development	10	106	3.52
immune system development	10	115	3.23
negative regulation of apoptosis	10	145	2.41
negative regulation of cell death	10	153	2.23
negative regulation of programmed cell death	10	145	2.41
**B. experienced *N. bryanti***			
metabolic process	96	2711	2.39
small molecule metabolic process	35	782	2.51
response to chemical stimulus	25	533	2.3
cellular ketone metabolic process	23	332	4.23
carboxylic acid metabolic process	22	321	4.07
organic acid metabolic process	22	328	3.96
oxoacid metabolic process	22	321	4.07
amine metabolic process	13	203	2.81
cellular amine metabolic process	12	181	2.82
cellular amino acid metabolic process	11	152	3.02
monocarboxylic acid metabolic process	10	157	2.43

“List” indicates the number of genes highly expressed within the term; “Gene set” indicates the total number of genes in that ontology included on the array.

### Differential expression between experienced woodrats of two species

The experienced *N. bryanti* had increased expression of more transcripts than *N. lepida* (134 vs 109) as well as a greater degree to which those genes were expressed (Additional file 4: Table S3). This result was also true with respect to the biotransformation transcripts alone; *N. bryanti* had greater expression of 3× more transcripts compared to *N. lepida* on creosote and, in general, these genes were expressed to a much higher degree (Table 2B and Table 5). The effective gene numbers also reflect this pattern. The effective gene number with greater expression in *N. bryanti* was 20.7, whereas the effective gene number for *N. lepida* was 14.4.

**Table 5:** **Transcripts associated with detoxification function with significantly greater expression in (A.) experienced *N. bryanti *** **relative to *N. lepida *** **and (B.) experienced *N. lepida *** **relative to *N. bryanti***

	**Gene ID**	**Gene Description**	**Ratio**	**p-value**
**A.**	Greater expression in experienced *N. bryanti*			
		**Phase I - catalyze oxidation**, **reduction & hydrolysis reactions**		
	**Aldehyde dehydrogenases - oxidize aldehydes**			
	NM_178713	Aldehyde dehydrogenase 8 family, member A1	2.22	0.010400
	**Alcohol dehydrogenases - oxidize alcohols**			
	NM_017270	Alcohol dehydrogenase 4 (class II), pi polypeptide	3.03	0.005872
	NM_017270	Alcohol dehydrogenase 4 (class II), pi polypeptide	2.82	0.005802
	NM_017270	Alcohol dehydrogenase 4 (class II), pi polypeptide	2.32	0.000857
	NM_017270	Alcohol dehydrogenase 4 (class II), pi polypeptide	2.26	0.002058
	**Carboxylesterases - hydrolyze carboxylic acid esters**			
	NM_001190346	Carboxylesterase 2 transcript variant 2, SimilarTo	2.61	0.003223
	XR_033674	PREDICTED: similar to Carboxylesterase 2	2.28	0.000942
	NM_001190346	Carboxylesterase 2 transcript variant 2. SimilarTo	2.24	0.002730
	NM_001190346	Carboxylesterase 2 transcript variant 2. SimilarTo	2.04	0.021685
	**Cytochromes P450 - oxidize wide range of organic substrates**			
	NM_008898	P450 (cytochrome) oxidoreductase	2.36	0.013012
	NM_147206	Cytochrome P450, family 3, subfamily a, polypeptide 9	2.18	0.021999
	NM_147206	Cytochrome P450, family 3, subfamily a, polypeptide 9	2.09	0.023839
	NM_153312	Cytochrome P450, family 3, subfamily a, polypeptide 23/polypeptide 1	2.08	0.014854
	**Flavin containing monooxygenases - oxidize amines**			
	NM_008030	Flavin containing monooxygenase 3	2.52	0.004188
	**Superoxide dismutases - dismutase superoxide to oxygen and water**			
	NM_017050	Superoxide dismutase 1	2.17	0.007301
		**Phase II - catalyze transfer of conjugates to metabolites**		
	**UDP glucuronosyltransferases - transfer glucuronic acid conjugate**			
	NM_152811	UDP glucuronosyltransferase 2 family, polypeptide B1	26.84	0.000202
	NM_152811	UDP glucuronosyltransferase 2 family, polypeptide B1	26.2	0.000230
	NM_153598	UDP glucuronosyltransferase 2 family, polypeptide B34	13.03	0.000721
	NM_153598	UDP glucuronosyltransferase 2 family, polypeptide B34	10.82	0.001924
	NM_153598	UDP glucuronosyltransferase 2 family, polypeptide B34	8.52	0.000934
	NM_152811	UDP glucuronosyltransferase 2 family, polypeptide B1	7.33	0.000471
	NM_152811	UDP glucuronosyltransferase 2 family, polypeptide B1	7.05	0.001293
	NM_153598	UDP glucuronosyltransferase 2 family, polypeptide B34	4.58	0.003637
	NM_173295	UDP glucuronosyltransferase 2 family, polypeptide B17	4.23	0.004670
	NM_173295	UDP glucuronosyltransferase 2 family, polypeptide B17	4.16	0.004114
	NM_173295	UDP glucuronosyltransferase 2 family, polypeptide B17	3.82	0.006675
	NM_153598	UDP glucuronosyltransferase 2 family, polypeptide B34	3.79	0.003846
	NM_153598	UDP glucuronosyltransferase 2 family, polypeptide B34	3.2	0.000542
	NM_153598	UDP glucuronosyltransferase 2 family, polypeptide B34	3.13	0.000852
	NM_001191676	UDP glucuronosyltransferase 2 polypeptide B34, SimilarTo	2.8	0.003254
	NM_153598	UDP glucuronosyltransferase 2 family, polypeptide B34	2.64	0.001950
	NM_001191676	UDP glucuronosyltransferase 2 family polypeptide B34	2.52	0.010507
	NM_201642	UDP glucuronosyltransferase 1 family, polypeptide A6B	2.34	0.009381
	NM_009467	UDP glucuronosyltransferase 2 family, polypeptide B5	2.23	0.003903
	NM_201642	UDP glucuronosyltransferase 1 family, polypeptide A6B	2.23	0.005852
	**Glutathione S-transferases - transfer glutathione conjugate**			
	NM_012796	Glutathione S-transferase, theta 2	5.9	0.002555
	NM_012796	Glutathione S-transferase, theta 2	4.93	0.003108
	NM_012796	Glutathione S-transferase, theta 2	4.79	0.003690
	NM_001024361	Similar to Glutathione S-transferase A1	3.98	0.005397
	NM_001024361	Similar to Glutathione S-transferase A1	3.58	0.008732
	NM_001024361	Similar to Glutathione S-transferase A1	3.52	0.005938
	NM_001024361	Similar to Glutathione S-transferase A1	3.25	0.014456
	NM_012796	Glutathione S-transferase, theta 2	2.89	0.032248
	NM_001024361	Similar to Glutathione S-transferase A1	2.66	0.008524
	NM_001024361	Similar to Glutathione S-transferase A1	2.39	0.010509
	NM_008183	Glutathione S-transferase, mu 2	2.34	0.047800
	NM_001009920	Glutathione S-transferase Yc2 subunit	2.32	0.018875
	NM_001009920	Glutathione S-transferase Yc2 subunit	2.1	0.025697
	NM_001077353	Glutathione S-transferase, alpha 3, transcript variant 2	2.08	0.048535
	**Methyltransferases - transfer methyl group conjugate**			
	NM_009349	Indolethylamine N-methyltransferase	10.85	0.000877
	NM_009349	Indolethylamine N-methyltransferase	8.38	0.002392
	NM_009349	Indolethylamine N-methyltransferase	6.52	0.009050
	NM_009349	Indolethylamine N-methyltransferase	6.47	0.007757
	NM_080462	Histamine N-methyltransferase	2.13	0.004838
	XM_223974	Methyltransferase 11 domain containing 1	2.04	0.005653
	NM_172687	Coenzyme Q3 homolog, methyltransferase (yeast)	2.03	0.008959
	**Sulfotransferases - tranfer sulfo group conjugate**			
	NM_020565	Sulfotransferase family 3A, member 1	4.17	0.006575
	NM_020565	Sulfotransferase family 3A, member 1	3.81	0.001338
	NM_020565	Sulfotransferase family 3A, member 1	3.52	0.007965
	NM_018805	Heparan sulfate (glucosamine) 3-O-sulfotransferase 3B1	3.22	0.001553
	NM_020565	Sulfotransferase family 3A, member 1	3.2	0.000616
	NM_020565	Sulfotransferase family 3A, member 1	2.83	0.004497
	NM_031834	Sulfotransferase family, cytosolic, 1A, phenol-preferring, member 1	2.79	0.001314
	NM_020565	Sulfotransferase family 3A, member 1	2.77	0.006085
	NM_018805	Heparan sulfate (glucosamine) 3-O-sulfotransferase 3B1	2.75	0.000909
	NM_020565	Sulfotransferase family 3A, member 1	2.53	0.000740
	NM_031834	Sulfotransferase family, cytosolic, 1A, phenol-preferring, member 1	2.26	0.003965
	NM_031834	Sulfotransferase family, cytosolic, 1A, phenol-preferring, member 1	2.05	0.004712
	NM_020565	Sulfotransferase family 3A, member 1	2.04	0.024154
**B.**	Greater expression in experienced *N. lepida*			
		**Phase I - catalyze oxidation**, **reduction & hydrolysis reactions**		
	**Aldehyde dehydrogenases - oxidize aldehydes**			
	NM_027406	Aldehyde dehydrogenase 1 family, member L1	9.62	0.030847
	NM_027406	Aldehyde dehydrogenase 1 family, member L1	4.11	0.045209
	**Alcohol dehydrogenases - oxidize alcohols**			
	NM_007410	Alcohol dehydrogenase 5 (class III), chi polypeptide	2.14	0.025440
	**Cytochromes P450 - oxidize wide range of organic substrates**			
	NM_028191	Cytochrome P450, family 2, subfamily c, polypeptide 65	8.87	0.017817
	NM_007820	Cytochrome P450, family 3, subfamily a, polypeptide 16	3.69	0.006413
	NM_019138	Cytochrome P450, family 7, subfamily b, polypeptide 1	2.68	0.015580
	NM_007815	Cytochrome P450, family 2, subfamily c, polypeptide 29	2.53	0.028674
	NM_023025	Cytochrome P450, family 2, subfamily J, polypeptide 4	2.45	0.004241
	NM_010009	25-hydroxyvitamin D3 1alpha-hydroxylase	2.37	0.030007
		**Phase II - catalyze transfer of conjugates to metabolites**		
	**Glutathione S-transferases - transfer glutathione conjugate**			
	NM_010358	Glutathione S-transferase, mu 1*	2.99	0.021783
	NM_010358	Glutathione S-transferase, mu 1*	2.94	0.009552
	NM_017013	Glutathione S-transferase A2	2.38	0.007870
	NM_017013	Glutathione S-transferase A2	2.34	0.010162
	NM_017013	Glutathione S-transferase A2	2.29	0.010172
	**Methyltransferases - transfer methyl group conjugate**			
	NM_030241	SET domain containing (lysine methyltransferase) 8	2.19	0.001281
	NM_025907	Methyltransferase like 6	2.05	0.019380
	NM_016668	Betaine-homocysteine methyltransferase	2.04	0.013408
	**Sulfotransferases - tranfer sulfo group conjugate**			
	NM_001101534	CDNA clone IMAGE:9053718	2.21	0.007280
	NM_027928	Carbohydrate (chondroitin 4) sulfotransferase 13, MutualBestHitTo	2.07	0.006258
	NM_027928	Carbohydrate (chondroitin 4) sulfotransferase 13, MutualBestHitTo	2.04	0.006510
	NM_027928	Carbohydrate (chondroitin 4) sulfotransferase 13, MutualBestHitTo	2.01	0.009812

Within species, results are organized by major detoxification enzyme classes. Duplicates indicate the response of multiple probes for a given gene.

In experienced *N. bryanti*, transcripts with the greatest expression were related to conjugation of metabolites with glucuronic acid (different glucuronosyltransferases) followed by those related to conjugation with glutathione (glutathione S-transferases; Table 5). In *N. lepida*, the transcripts with the highest expression were those related to functionalization of aldehydes by aldehyde dehydrogenase (1 L1) and oxidation by Cytochrome P450 (2C65). Compared to *N. bryanti*, *N. lepida* also had greater expression of some of the same transcripts that were significantly induced by creosote feeding, i.e., aldehyde dehydrogenase (1 L1) glutathione S-transferase mu-1 (Tables 1 and 5).

Significant GO terms overrepresented by experienced *N. bryanti* fed creosote were characterized by metabolic and catabolic processes as well as responses to stimuli (Table 6A). GO terms overrepresented by experienced *N. lepida* are characterized by responses to stimuli and signaling functions (Table 6B).

**Table 6:** **Gene ontology** (**GO**) **terms overrepresented in (A) *N. bryanti *** **and** (B) ***N. lepida*** **on a creosote diet**

**Ontology**	**List**	**Gene set**	**z-score**
**A**. ***experienced N. bryanti***			
metabolic process	65	2711	2.58
response to stimulus	36	1241	2.73
small molecule metabolic process	30	782	4.09
cellular response to stimulus	26	882	2.28
catabolic process	24	626	3.57
cellular catabolic process	21	550	3.28
response to chemical stimulus	18	533	2.44
oxidation-reduction process	17	375	3.68
cellular ketone metabolic process	15	332	3.42
carboxylic acid metabolic process	14	321	3.15
organic acid metabolic process	14	328	3.06
oxoacid metabolic process	14	321	3.15
response to organic substance	14	348	2.82
small molecule catabolic process	13	282	3.25
lipid metabolic process	12	316	2.38
cellular response to chemical stimulus	10	222	2.74
response to endogenous stimulus	10	186	3.37
**B. experienced *N. lepida***			
response to stimulus	35	1241	2.13
Signaling	25	764	2.52
signal transduction	24	689	2.79
regulation of response to stimulus	16	430	2.49
intracellular signal transduction	15	347	3.03
oxidation-reduction process	15	375	2.7
regulation of signal transduction	15	327	3.28
regulation of signaling	15	363	2.84
lipid metabolic process	13	316	2.61

“List” indicates the number of genes highly expressed within the term; “Gene set” indicates the total number of genes in that ontology included on the array.

## Discussion

Despite the vast knowledge of drug-metabolizing enzymes in humans and model species, the biotransformation mechanisms used by mammalian herbivores to metabolize PSCs are largely unknown. Until recently, many studies were limited to analysis of one or a few biotransformation enzymes [32,37-40]. This study took advantage of microarray technology customized for a unique study system to address this deficit in our understanding. We investigated, on a transcriptomic scale, the genes induced by a particular suite of PSCs (creosote resin) and evaluated whether a common set of genes were expressed by herbivores with varying levels of evolutionary experience with these PSCs. We explored whether experienced herbivores have independently converged on regulation of a similar set of biotransformation genes. We found that biotransformation enzyme expression does indeed vary with ecological and evolutionary experience with creosote and that independent woodrat lineages employ, in part, similar strategies for successfully dealing with these shared PSCs. In addition, the results narrow the field from hundreds of possible biotransformation genes to less than ten candidates. The work provides a testable framework for the changes in expression of biotransformation enzymes that may have occurred as woodrats shifted from one toxic diet to another.

### Induced biotransformation genes

We identified a narrow set of candidate genes relevant to the biotransformation of creosote. Surprisingly few biotransformation transcripts were induced by any of the groups fed creosote resin compared to the control diet. Of the hundreds of biotransformation enzymes, only four unique biotransformation transcripts were induced in the naïve *N. bryanti*, six in the experienced *N. lepida*, and in the experienced *N. bryanti*, only a single biotransformation enzyme, an aldo-keto reductase (AKR7A3) was induced (Table 1). All of these transcripts encode for enzymes that act on substrates similar to the compounds present in creosote, particularly aromatic compounds [27,31]. These enzymes could function in tandem as a pathway to produce the final metabolite excreted in urine and/or feces [41]. For example, epoxide hydrolase acts on aromatic compounds such as naphthalene epoxide whose parent compound, napthalene, is present in creosote [41]. AKR enzymes, in turn, act on the metabolites produced by epoxide hydrolase, whereas glutathione S-transferases and UDP glucuronosyltransferases add conjugates to the metabolites of AKR [42]. Thus, although few transcripts were induced in woodrats fed creosote, those induced transcripts produce enzymes that act on substrates similar to those in resin. Furthermore, the candidate genes identified could function in concert with one another in the biotransformation of PSCs in creosote.

Few of the transcripts induced by the creosote diet were shared across woodrat groups. The conspecific populations shared induction of AKR7A3. The superfamily of AKR enzymes act on a broad variety of substrates, particularly aldehydes and ketones, and the 7A3 isoform metabolizes aflatoxin B1 [42]. Epoxide hydrolase was induced by the naïve *N. bryanti* and experienced *N. lepida*, and also the experienced *N. bryanti* but in this case, at less than the 2-fold cut-off. This enzyme adds water to epoxides that could otherwise cause toxicity or mutation [31]. Epoxides are often formed during the biotransformation of aromatic hydrocarbons [31], which are common in creosote resin [27]. The shared induction across all three groups of woodrats suggests epoxide hydrolase could be critical to the biotransformation of resin. Lastly, the naïve *N. bryanti* and experienced *N. lepida* induced different sulfotransferases. This superfamily has high affinity to myriad substrates and metabolizes compounds (e.g., polyaromatic hydrocarbons) present in creosote resin [31]. Sulfotransferases biotransform xenobiotics by the addition of a sulfur co-factor, generated from dietary cysteine. Because the availability of cysteine is often limited, sulfation capacity is often much lower than other conjugation pathways. It is possible that the woodrat diet contains ample cysteine or its precursors, serine and methionine. Consistent with this idea, both experienced populations upregulated methyltransferases involved in serine and methionine metabolism (Tables 3 and 5). Alternatively, sulfation capacity may be greater in woodrats compared to other mammals. However, previous work suggests the capacity for sulfation is exceeded in both naïve and experienced *N. lepida* at low levels of creosote ingestion [43]. The role that sulfation plays in the biotransformation of creosote resin deserves further attention.

### Alterations in biotransformation mechanisms as a result of diet shifts

The change by woodrats from an ancestral diet of juniper and/or cactus to that of creosote likely required a different set of biotransformation enzymes to process the disparate suites of PSCs in these plants. The naïve and experienced *N. bryanti* had increased expression of about the same number of genes on creosote compared to the control diet. However, within that pool of genes, the naïve *N. bryanti* expressed fewer transcripts with biotransformation functions than the experienced animals. A similar pattern has been observed elsewhere; woodrats feeding on a novel diet (i.e., animals naïve to the diet) expressed relatively few biotransformation transcripts and more transcripts related to cellular function compared to feeding on their native diet [33]. The animals on the novel diet were thought to be unable to marshal the appropriate biotransformation response, and the upregulation of genes with diverse cellular functions may have been to prevent or respond to the physiological consequences of the PSCs. Indeed, the functional analyses of the gene ontologies for differentially expressed genes in *N. bryanti* are consistent with this hypothesis (Table 4). The transcriptomes of naïve woodrats were enriched in GO classes related to considerable physiological stresses (e.g., death, response to wounding). In contrast, those enriched in experienced *N. bryanti* were related to the processing of toxins.

### Functional convergence

There is evidence for functional convergence between the two species with respect to the transcripts induced on a creosote diet. As discussed above, the transcript for epoxide hydrolase was the only biotransformation transcript significantly induced by both species; however, it was expressed at levels below the 2-fold cut-off used in the initial analysis. The top biotransformation transcript induced by *N. lepida* (aldehyde dehydrogenase) and the only biotransformation transcript induced by *N. bryanti* (aldo-keto reductase) both encode for enzymes that metabolize similar substrates, particularly aldehydes. Creosote resin contains at least 300 compounds, including aldehydes and compounds that may have aldehyde functional groups after biotransformation by other enzymes [27,44]. Aldehydes are reactive compounds that cause considerable cellular damage. Inadequacies in the biotransformation of aldehydes are connected to a number of human diseases [45,46]. Given the potential for damage, aldehydes in creosote are likely a significant selective force resulting in the convergence on the induction of transcripts related to aldehyde metabolism by both species.

Further demonstration that the two experienced woodrats exhibit a parallel response to the ingestion of creosote resin is witnessed in their overall gene expression patterns. Many of the biotransformation transcripts that were more highly expressed in the experienced *N. bryanti* have analogous functions with those that were more highly expressed in *N. lepida* (Table 5). For example, both species have higher expression of different isoforms of aldehyde dehydrogenases (ALDH8A1 versus ALDH1L1). A similar pattern is present for alcohol dehydrogenases, Cytochromes P450, methyltransferases, glutathione S-transferases, and sulfotransferases. One notable exception to this pattern is that *N. lepida* did not have a corollary in the UDP-glucuronosyltransferases (UGTs), a superfamily of biotransformation enzymes responsible for glucuronidation. *Neotoma bryanti* had higher expression of at least 6 different UGTs, one of which was expressed 26 fold higher. The UGTs are considered some of the most versatile of the conjugation enzymes due to their wide range of substrates [31]. Previous studies reported that *N. lepida* fed creosote increased glucuronidation thereby demonstrating it is indeed an important pathway in *N. lepida*[32,43]. It is possible that both species use the same UGTs for creosote biotransformation but that *N. bryanti* utilizes this pathway to a greater extent resulting in the higher gene expression values observed in this study.

The last line of evidence for convergence stems from GO results. The experienced populations fed creosote share three ontologies (response to stimulus, oxidation-reduction process, lipid metabolic process), which all relate to biotransformation (Table 6).

### Constitutive differences

While many biotransformation enzymes are induced when an animal is exposed to xenobiotics, some are expressed constitutively at high levels [31]. It is difficult to identify such baseline differences in expression using microarrays. For example, a recent study on an herbivorous insect that specializes on glucosinolate-rich plants did not identify any induced biotransformation transcripts when the insects were fed glucosinolates compared to a control diet [47]. Constitutive expression could explain this outcome. In our study, direct comparisons of gene expression between populations fed creosote provide insight into how animals differ in baseline biotransformation, especially when coupled with the results from the induction study. The experienced *N. bryanti* induce very few transcripts, only one of which has known biotransformation function, relative to both the naïve *N. bryanti* and the experienced *N. lepida*. However, direct comparisons of experienced *N. bryanti* with both the naïve conspecific and experienced congeneric when all are fed creosote, revealed many more differences in expression (Table 2A and B). Overall, the experienced *N. bryanti* had greater expression of significantly more biotransformation enzymes. This pattern coupled with the paucity of induced transcripts suggests that the experienced *N. bryanti* may be constitutively expressing biotransformation enzymes at a higher level. It is possible that *N. bryanti*’s longer historic exposure to creosote caused an increase in baseline production of biotransformation enzymes such that they continually express the appropriate combination of enzymes to efficiently detoxify creosote PSCs [22,23]. Moreover, the experienced *N. lepida* GO terms include many for regulation and signal transduction, suggesting processes in flux, whereas these terms are absent from the experienced *N. bryanti* results (Table 6).

## Conclusions

Herbivores and the plants on which they feed represent one of the most common interactions in nature, yet we know relatively little about the mechanisms that herbivores, particularly mammalian ones, employ to overcome plant toxins [1,48]. The application of recently developed genome-based approaches is enabling researchers to more thoroughly investigate the responses of herbivores to plant defensive compounds. This work capitalized on the availability of a recently developed microarray to identify the genes induced by mammalian herbivores when feeding on PSCs. The results lay the requisite groundwork for future functional and evolutionary studies of the genes involved in the metabolism of creosote toxins and the evolution of diet switching in woodrats.

## Methods

### Trapping and feeding trial

We trapped two species, *N. lepida* (desert woodrat) and *N. bryanti* (Bryant’s woodrat). *Neotoma lepida* were trapped at Lytle Ranch Preserve (lat: 37.117514, long: -114.009661, Washington Co., UT, USA). *Neotoma bryanti* were trapped near Palm Springs (lat: 33.679616 long: -116.362018, Riverside Co., CA, USA). These two populations were chosen because both the desert woodrat and Bryant’s woodrat at these sites include creosote bush (*L. tridentata*) as a primary component of their diet [35] (unpublished observations by MDD and JRM). With respect to creosote ingestion, we refer to these populations as “experienced,” both in their individual histories as well as their evolutionary histories. In preliminary trials, experienced animals from both species were capable of ingesting high levels of creosote resin (8% by dry mass) added to a powdered rabbit chow base (Teklad formula 2031). In addition, we trapped *N. bryanti* at Ronald W. Caspers Wilderness Park (lat: 33.53367, long: -117.54965, Orange Co., CA, USA). This population has no evolutionary or ecological exposure to creosote bush, and is considered “naïve” to creosote PSCs. In preliminary trials, these animals did not maintain body mass on diets of 4% creosote resin. All experimental procedures were approved by the University of Utah’s Institutional Animal Care and Use Committee (#07-02015).

Eight woodrats from each of the three groups (experienced *N. lepida*, experienced *N. bryanti*, naïve *N. bryanti*) were divided into two treatments of four individuals (n = 2 males, 2 females). One treatment was fed powdered rabbit chow amended with resin from creosote bush; the other group was fed a control diet of the same powdered rabbit chow with no additions. Resin was extracted and creosote diets prepared as in [34].

Animals given the resin diet were fed a gradually increasing amount of resin over an eight-day period to allow induction of biotransformation enzymes [31]. This group received powdered chow with 0% resin for three days, 1% creosote resin (dw/dw) for two days, followed by 2% creosote resin for three days. The final 2% concentration was selected to accommodate the naïve *N. bryanti* since they were unable to tolerate more resin without excessive mass loss (>10% initial weight). Animals in the control treatments received 0% resin diet for all eight days. Animals had water ad libitum, and diet was presented daily. Animals were weighed daily and body masses were compared within and between groups using a repeated measures ANOVA with time as the repeated variable and population and diet as factors.

Animals were dispatched using CO_2_ asphyxiation; liver tissue was preserved in RNAlater (Sigma) and archived at -80°C. RNA was extracted (RNAqueous) for the microarray analysis.

### Microarray analysis

Liver samples were analyzed using a custom microarray, built from the hepatic transcriptome of a single desert woodrat [36]. Two primary probe groups were designed from the transcriptome. The target contigs for the first group (n = 943) were woodrat contigs whose annotation matched a list of biotransformation genes extracted from Norway rat arrays that we had previously used in studies with woodrats [34,49]. These probes were all labeled “biotransformation.” The target contigs in the second group consisted of contigs from the woodrat transcriptome that had ≥75% sequence identity with a rodent BLAST match in the region of highest homology (n = 5355). All probes were screened for quality and replicated on the array.

The RNA quality and concentration of each sample was evaluated, and the Agilent One-Color Quick Amp Labeling Kit was used to generate fluorescently labeled cRNA for hybridizations. Additional details on microarray processing methods are available in [36]. Microarray features were extracted using Agilent Feature Extraction software version 10.5.1.1. All control spots, non-uniform spots and population outlier spots were removed from the dataset; intensity values were log^2^ transformed using AgilentFilter, software designed to simplify the processing of Agilent data. Data from duplicate probes were combined, but different probes with the same annotation were maintained separately since it is possible that the original contigs were from different isoforms despite the shared match to a rodent gene. Consequently, many of the resulting lists of differentially expressed genes have multiple seemingly identical entries. For most analyses, we treat these probes as targeting unique genes; in a few specified analyses, we have combined or discarded duplicate probes to evaluate the results as conservatively as possible. The performance of the custom *Neotoma* expression array was previously validated [36].

### Species and diet comparisons

To compare gene expression across diet and species, the data for the *Neotoma* probes was batch uploaded to Genesifter 3.7. Prior to comparing expression profiles, the consistency of transcriptome response was evaluated by comparing overall gene expression profiles across individuals. After normalizing the intensity data, individuals were clustered by gene expression using all woodrat-derived probes (n = 6286). Clustering parameters were distance:correlation, linkage:average, row centered:by genes.

We created a total of five pairwise comparisons in Genesifter. The specific nature of these comparisons are described in the subsequent paragraphs. In all comparisons, the quality requirement was set to 1, and all individuals from both treatments were required to pass. T-tests were performed with alpha = 0.05, and resulting lists of significantly differentially expressed genes/probes were ordered by fold change with a fold change threshold of greater or equal to 2. This approach is less conservative than implementing a statistical control for the false discovery rate (e.g., Benjamini and Hochberg correction [50]), but fold change ranking is more consistent [51].

To identify induction and repression of genes with respect to ingestion of resin, we compared expression on the creosote diet to the control diet for each of the three groups (experienced *N. lepida*, experienced *N. bryanti*, naïve *N. bryanti*). The lists of significantly differently expressed genes were ordered by direction and fold change. Transcripts that were upregulated on the creosote diet were considered induced by creosote and may be indicative of enzymes important in the biotransformation of creosote. Transcripts that were downregulated were considered repressed. To test for conserved or convergent responses, the lists of induced genes were compared for entries present in more than one woodrat group.

In the fourth comparison, naïve and experienced *N. bryanti* fed the creosote diet, were compared to identify contrasts in expression with respect to prior experience. Using the gene lists generated from these comparisons, we compared the overall number of transcripts with greater expression in each group as well as the subset of these transcripts characterized by having biotransformation function. The standard gene lists treat probe entries with identical Genbank accession numbers as independent listings. To more conservatively compare the relative response of each group to the creosote diet, we quantified the number of unique Genbank accessions in the biotransformation gene lists and, then calculated the Shannon’s H Diversity Index from each list. To calculate Shannon’s H, each unique Genbank accession was treated as a “species” and the number of times it appeared in the significantly upregulated list was treated as its “abundance”. The values for Shannon’s H were then used to calculate an effective number of upregulated genes i.e., the number of “species” expected in comparable communities given their Shannon’s H. This index is used to contextualize diversity indices across multiple communities [52]. The effective species value (or effective gene value, in this case) is calculated as exp (H).

To examine function more broadly, we used Gene Ontology (GO). GO is a bioinformatics tool that groups individual genes by the function of their products. GO allows the organization of many individual gene results into fewer functionally-defined categories. There are 3 major classifications: cellular component, molecular function and biological process. Within each of these three, function is categorized more and more specifically. Within our GO results, z-scores were used to determine significantly important associations. Ontology terms with z-scores of >2 are considered to be significantly overrepresented in the results; that is, the genes in that ontology are more likely to be upregulated than expected by chance, given the number features on the array within that particular GO term. Within the biological process classification, two gene ontology lists were generated for transcripts with greater expression in naïve *N. bryanti* and in experienced *N. bryanti*. Lastly, experienced animals from both species fed the creosote diet were compared to illuminate contrasts in expression between species with similar experience. This contrast was conducted in the same fashion as the naïve versus experienced *N. bryanti*.

## Abbreviations

PSC: Plant secondary compounds; NDGA: Nordihydroguiairetic acid; GO: Gene ontology; AKR: Aldo-keto reductase; UGT: UDP glucuronosyltransferase.

## Competing interests

The authors declare that they have no competing interests.

## Authors’ contributions

JRM: Performed the experiments, analyzed the data, contributed to manuscript draft; MSS: Contributed to the analysis and interpretation; MDD: Designed the study, obtained funding and contributed to manuscript draft, contributing equally with JRM. All authors contributed substantially to manuscript revisions. All authors read and approved the final manuscript.

## Supplementary Material

Additional file 1: Table S1 Animal masses before and after feeding trial. Click here for file

Additional file 2: Figure S1 Cluster analysis of individual woodrat samples labeled by treatment. Description: Branching pattern determined by overall gene expression patterns. Individual animals are labelled with species and experience with creosote bush in the wild; creosote diet in laboratory trial is indicated by bolded text. Click here for file

Additional file 3: Table S2 Significantly differently expressed transcripts from comparison of naïve versus experienced *N. bryanti*. Description: (A.) Transcripts with greater expression by naïve *N. bryanti*; (B.) transcripts with greater expression by experienced *N. bryanti*. All animals were fed a creosote diet. Duplicates indicate the response of multiple probes for a given gene. Click here for file

Additional file 4: Table S3 Significantly differently expressed transcripts from comparison of experience N. byranti versus experienced *N. lepida*. Description: (A.) Transcripts with greater expression by *N. bryanti*; (B.) transcripts with greater expression by *N. lepida*. All animals were fed a creosote diet. Duplicates indicate the response of multiple probes for a given gene. Click here for file
