# Supplementary material for: Evidence for functional convergence in genes upregulated by herbivores ingesting plant secondary compounds
Source: BMC Ecol. 2014 Aug 15;14:23. doi: 10.1186/1472-6785-14-23 (PMC4153740; doi:10.1186/1472-6785-14-23)

Additional Figure 1. Cluster analysis of individual woodrat samples labelled by treatment. Branching pattern determined by overall gene expression patterns. Individual animals are labelled with species and experience with creosote bush in the wild; creosote diet in laboratory trial is indicated by bolded text.

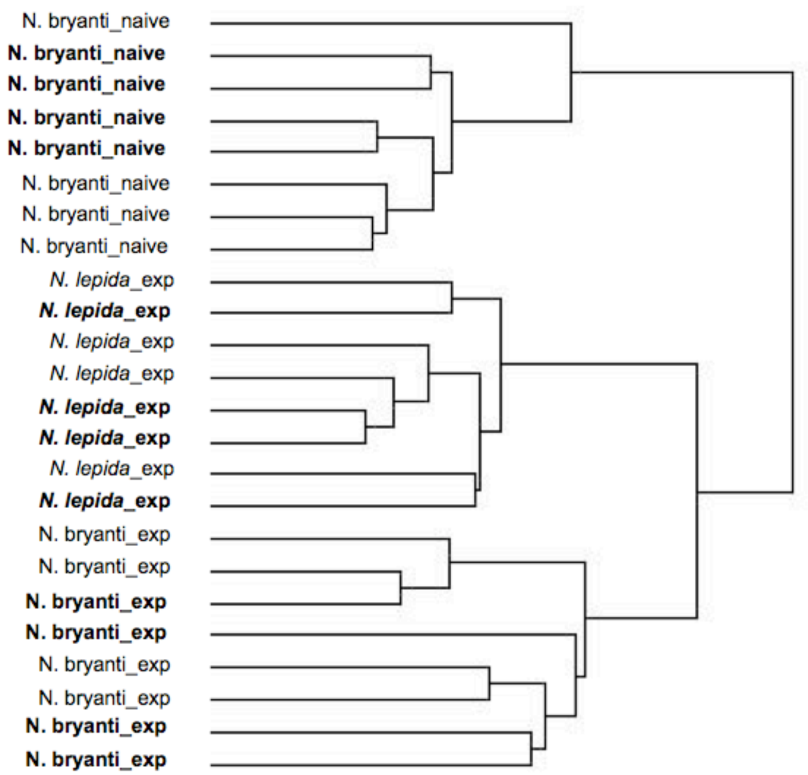

Supplement: Additional file 2: Figure S1 — Cluster analysis of individual woodrat samples labeled by treatment. Description: Branching pattern determined by overall gene expression patterns. Individual animals are labelled with species and experience with creosote bush in the wild; creosote diet in laboratory trial is indicated by bolded text. [file 1472-6785-14-23-S2.pdf]
